# Intrinsically disordered regions facilitate Mlp1–Nab2 recognition in mRNA quality control

**DOI:** 10.1080/19491034.2026.2680376

**Published:** 2026-06-03

**Authors:** Mohammad Soheilypour, Mohaddeseh Peyro, Hengameh Shams, Mohammad R. K. Mofrad

**Affiliations:** a Molecular Cell Biomechanics Laboratory, Departments of Bioengineering and Mechanical Engineering, University of California, Berkeley, CA, USA; b Molecular Biophysics and Integrative Bioimaging Division, Lawrence Berkeley National Lab, Berkeley, CA, USA

**Keywords:** intrinsically disordered proteins, molecular dynamics simulations, mRNA export, mRNA quality control, nuclear basket, nuclear pore complex (NPC), RNA-binding proteins (RBPs)

## Abstract

Quality control of mRNAs ensures that only properly processed transcripts are exported from the nucleus. Myosin-like protein 1 (Mlp1), plays a central role in this process by interacting with RNA-binding proteins (RBPs), including Nab2. While previous studies identified Phe73 in Nab2 as critical for Mlp1 binding, the molecular mechanism remains unclear. Here, we employed a computational approach to develop a mechanistic model of Mlp1–Nab2 interaction. Our results suggest that Phe73 does not act through direct contacts with Mlp1, but instead stabilizes intramolecular interactions between Nab2 helices that promote a compact conformation. F73A disrupted this helix–helix stabilization and weakened binding, whereas F73W enhanced the interaction. Our findings support a binding mechanism in which the structural flexibility of Mlp1’s disordered domain enables adaptive recognition of Nab2. This mechanism may represent a general strategy by which the nuclear basket inspects mRNPs, highlighting the importance of flexible protein–protein recognition in mRNA quality control.

## Introduction

Nuclear export of messenger RNA (mRNA) is an essential step in the regulation of gene expression in eukaryotic cells [1], ensuring that transcripts reach the cytoplasm for translation. In contrast to other RNA species (tRNA, miRNA, snRNA, rRNA) that are transported via a karyopherin (Kap)-mediated pathway, bulk of mRNA export is mediated by conserved heterodimeric export receptor Mex67-Mtr2 (TAP-p15 or NXF1-NXT1) [2]. While Kap-mediated export requires RanGTPase cycle for directionality, mRNA export does not directly depend on the RanGTPase system [3]. Transcription of mRNA is followed by several processing and packaging steps, eventually forming an export-competent messenger ribonucleoprotein (mRNP) to be exported to the cytoplasm through the nuclear pore complexes (NPCs) [4]. mRNAs are spliced and undergo 5’-end capping, 3’-end cleavage and polyadenylation processes inside the nucleus [5]. In addition, they are co-transcriptionally loaded with several RNA-binding proteins (RBPs) (such as Npl3 by RNA polymerase II and Gbp2 and Hrb1 via the THO complex), which play a key role in the transport of mRNAs [6–8].

Eukaryotic cells have evolved quality control mechanisms at multiple stages of mRNA biogenesis to ensure that only fully processed mRNAs are exported [9]. One critical quality checkpoint occurs at the entry of the NPC, where a network of RBPs and NPC-associated factors (including Nab2, Gbp2, Hrb1, Npl3, Mlp1, Mlp2, Nup60, Pml1, Pml39, and Esc1) scrutinize mRNPs before export [10]. Properly processed mRNAs, marked by the appropriate RBPs, are cleared for export by recruiting Mex67–Mtr2, whereas aberrant or incompletely processed mRNAs are retained inside the nucleus and will be marked by TRAMP (Trf4/Air2/Mtr4 Polyadenylation) complex for degradation at the nuclear exosome [11,12]. Cross-interactions between the export machinery and quality control factors ultimately recognize aberrant mRNAs and target them for nuclear decay, preventing translation of improper transcripts [13].

Among the NPC basket proteins, the myosin-like protein 1 (Mlp1) in yeast (Tpr in vertebrates) plays a central role in this mRNA quality control checkpoint [10,14]. While Mlp1 is not essential for cell viability [15,16], deletion of Mlp1 leads to impaired retention of intron-containing mRNA transcripts [17], underscoring Mlp1’s role in ensuring only properly processed mRNAs are exported. The role of Mlp1 in mRNA quality control is further supported by studies that demonstrate the interaction between Mlp1 and various RBPs, including Nab2 [18–20], Gbp2 and Hrb1 [13], and Npl3 [18]. Our previous computational modeling study on the overall dynamics of mRNA quality control also highlighted the significance of the interaction between RBPs and Mlp1 in mRNA quality control [21]. Mlp1 is suggested to act both as a quality control checkpoint and a docking site that transiently tethers mature mRNPs at the pore, enabling the complex to undergo the necessary remodeling steps [22].

Nab2, one of the key yeast RBPs, is a zinc finger protein essential for cell viability and plays multiple roles in mRNA processing and export. It is an essential nuclear RBP that is required for the export of poly(A) RNA transcripts [23,24]. Nab2 shuttles between the nucleus and the cytoplasm and its lifecycle is regulated via three functional domains [25] (28), namely the N-terminal domain (residues 1–97) essential for the export of mRNA and Nab2 into the cytoplasm [19,26], an RGG domain (residues 201–265) as a nuclear localization signal (NLS) for the import of Nab2 into the nucleus via Kap104 [27], and a zinc finger domain (residues 262–477) for specific binding to polyadenosine RNAs [19,26,28]. The N-terminal domain is essential for Nab2’s function in mRNA export, where deleting this domain results in nuclear accumulation of poly(A) RNA [26,29]. Notably, this N-terminal domain is the segment of Nab2 that engages with Mlp1 at the nuclear pore [23].

The interaction between Nab2 and Mlp1 has been the subject of several *in vivo* and *in vitro* studies. Initial work identified the C-terminal domain of Mlp1 as critical for Nab2 binding: specifically, a 385-residue region (residues 1490–1875) of Mlp1 was shown to bind the Nab2 N-terminus [18]. Nab2-N is both necessary and sufficient for this interaction [20]. However, a follow-up study refined the Nab2-binding domain of Mlp1 to a 183-residue region (Mlp1-C; residues 1586–1768) [19]. These studies identified a hydrophobic patch in Nab2 N-terminal domain, centered on Phe73, which was suggested as being critical for the interaction between the two proteins. However, the exact underlying molecular mechanism is still elusive, which limits the current understanding of mRNA quality control mechanisms.

We previously performed various computational studies, ranging from the exploration of atomic-scale interactions to the analysis of the overall dynamics of the system, as an attempt to shed light on different aspects of nucleocytoplasmic transport in general [30–32, 33] and mRNA export and quality control in particular [21,34,35]. Here, we employ an integrated computational approach to explore, in greater detail, the pivotal interaction between Mlp1 and Nab2 in mRNA export and quality control processes. First, we use structure prediction tools to define representative structural features of the Mlp1-C region. Subsequently, we perform docking simulations between Nab2-N and Mlp1-C to sample plausible binding orientations consistent with experimental constraints. Following some refinements, we perform molecular dynamics (MD) simulations to explore the detailed molecular mechanism of this interaction. Previous experiments showed that an F73A mutation in Nab2 disrupts Nab2–Mlp1 binding and mRNA export [18,19]. By comparing wild-type and mutant simulations, we aimed to pinpoint how Phe73 contributes to complex formation. Together, these simulations provide a mechanistic model for the Mlp1–Nab2 interaction that helps rationalize existing experimental observations and offers new insight into the role of the Mlp1–Nab2 checkpoint in mRNA export.

## Materials and methods

Molecular dynamics (MD) simulations were performed using GROMACS v4.5.3 and v5.0.1 [36] with the CHARMM27 force field [37,38]. Water and ion addition as well as all protein manipulations were done using Gromacs modules, with the exception of residue mutation, which was performed using VMD v1.9.1 [39]. The crystal structure of the N-terminal domain of Nab2 (PDB ID: 2V75) [20] was used directly in simulations. As no experimental structure of the C-terminal domain of Mlp1 is available, likely due to its partially disordered structure similar to the human homologue Tpr [40], we generated representative structural models using multiple secondary- and tertiary-structure prediction servers (Phyre2, I-TASSER, RaptorX, Psi-Pred, and HHpred) [41–45]. Protein–protein docking was performed using ZDOCK v3.0.2, as well as PatchDock and FireDock [46–48] to sample plausible interaction orientations between Mlp1-C and Nab2-N. Prior to docking, Nab2-N and Mlp1-C models were equilibrated for 20 ns in explicit solvent to relax potential modeling artifacts. The top 10 docking solutions were examined as candidate interaction modes. Since Phe73 was experimentally observed to be essential for this interaction, docking solutions in which the hydrophobic helix of Nab2 (where Phe73 is located) was not oriented toward Mlp1-C (solutions 2, 4, 5, 9, and 10) were excluded.

Each system was solvated in a TIP3P water box with a 22A margin, neutralized with counter-ions, and the ion concentration was set to 150 mM NaCl. Periodic boundary conditions were applied. Energy minimization was performed for 5,000 steps with a 2 fs timestep, followed by 10 ns MD runs (1 ns equilibration, 9 ns production). For extended simulations, because Mlp1-C contains a substantial intrinsically disordered region, each wild-type and mutant complex was equilibrated for 100 ns to allow adequate sampling of conformational space, followed by an additional 20 ns production run. Each simulation was repeated three times to account for stochastic variation. Data visualization and dynamic cross-correlation analyses were performed using the Bio3D R package [49], and molecular graphics were generated with VMD v1.9.1. Inter-helical distances in Nab2-N were calculated as the center-of-mass distance between helix4 and helix5.

## Results

### Constructing the Mlp1–Nab2 complex

Since no experimental structure is available for the Nab2-binding domain of Mlp1-C, we used various secondary- and tertiary-structure prediction tools, including Phyre2, I-TASSER, RaptorX, PsiPred, and HHpred [1–5], to obtain representative structural models of Mlp1-C. Across all methods, there was strong agreement that Mlp1-C comprises a helical segment followed by a predominantly flexible and intrinsically disordered C-terminal domain, although the precise conformation of this disordered portion cannot be defined (Supplementary Materials). This overall organization is consistent with previous reports on the human homologue Tpr, which contains a long coiled-coil region followed by a disordered C-terminal domain [7]. Accordingly, we used the Phyre2 model as a representative starting conformation of Mlp1-C for docking and MD simulations against the experimentally determined Nab2-N structure (Figure S1).

**Figure 1.**
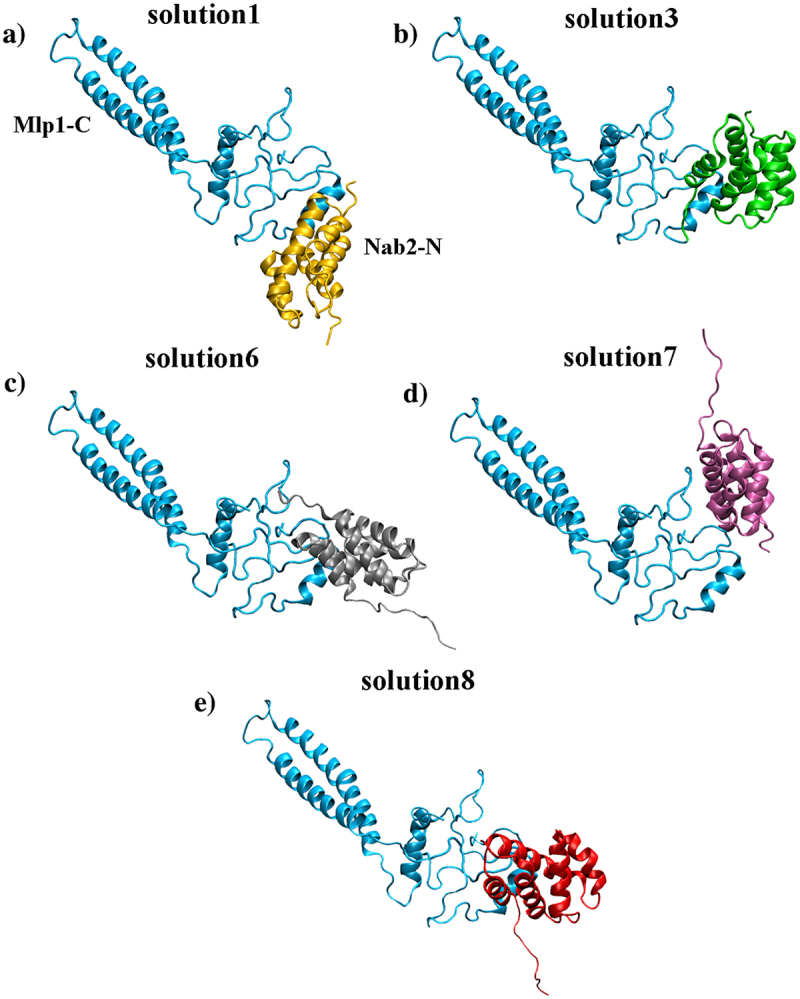
Representative binding orientations for Mlp1-C:Nab2-N complex. The five docking solutions shown are those consistent with experimental evidence indicating a critical role for Phe73 in the interaction. Mlp1-C is shown in blue.

Since no structural information is available for the Mlp1–Nab2 complex, we used molecular docking (ZDOCK [8]) to explore a range of plausible interaction modes consistent with available experimental constraints. Across the top-ranked docking poses, Nab2-N was rarely positioned near the structured helical segment of Mlp1-C, suggesting that this region is unlikely to serve as the primary docking site (Figure S2). The top 10 ZDOCK solutions were examined as plausible orientations of the Mlp1-C:Nab2-N complex. Previous studies identified a hydrophobic patch in Nab2-N centered on Phe73 as essential for the interaction with Mlp1-C [18,19]. Accordingly, docking solutions in which Phe73 was not oriented toward Mlp1-C were considered inconsistent with available experimental observations and were excluded. The remaining five orientations are shown in Figure 1.

**Figure 2.**
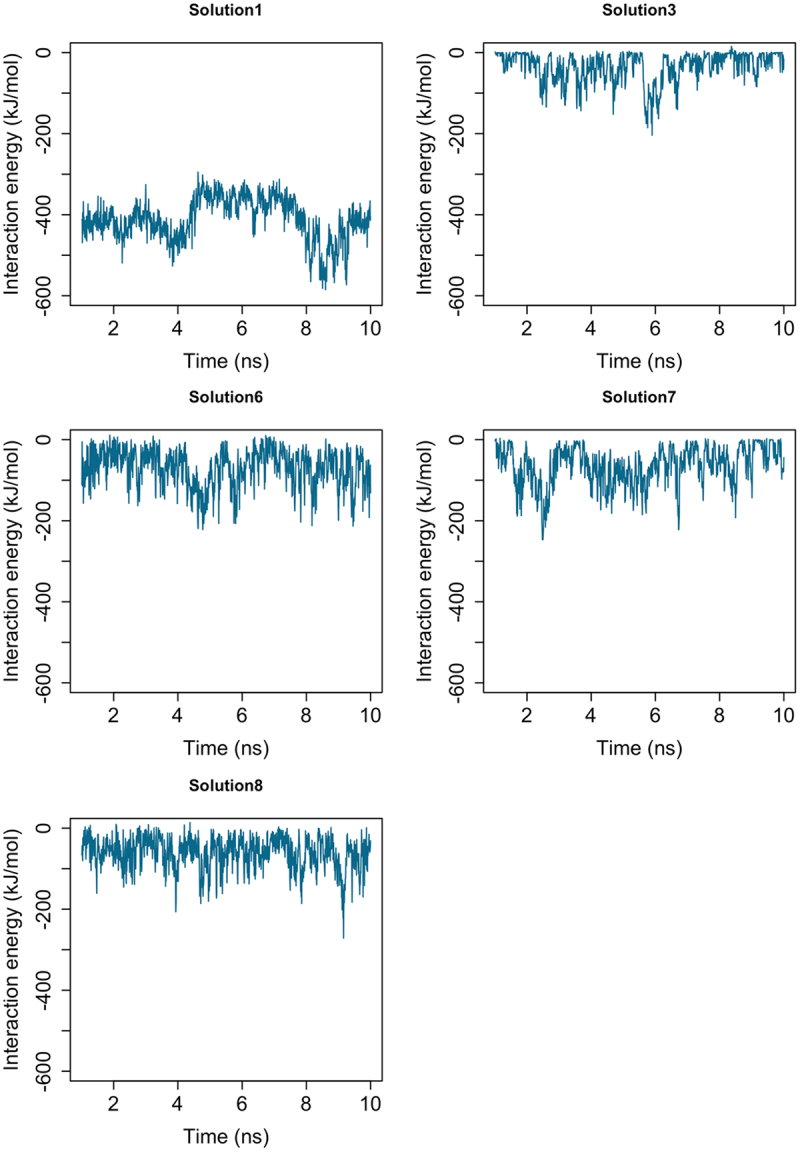
Interaction energies between Mlp1-C and Nab2-N for the five selected docking orientations after 10 ns of MD simulation following equilibration. Among the samples configurations, solution 1 exhibits a consistently stronger interaction energy relative to the other orientations.

While docking provided a set of plausible binding poses, it does not account for the inherent flexibility of the Mlp1-C disordered region or provide information about the relative stability of each pose. In order to identify the most favorable orientation for binding, we performed MD simulations of each of the five complexes (Figure 1). Each system was simulated for 10 ns, excluding the first nanosecond as equilibration. Interaction energies between Nab2-N and Mlp1-C were monitored (Figure 2). Among the solutions, orientation 1 consistently showed stronger interaction energy than the other poses and was therefore selected as the most favorable among the sampled configurations for further analysis. To confirm this result, we repeated docking using the PatchDock [46] and FireDock [47] servers. Out of the top 10 solutions (Figure S5), nine were inconsistent with the orientation of Phe73. The remaining solution (solution 8) closely matched solution 1 from ZDOCK, further validating this configuration as a representative binding mode (Figure S6).

### Phe73 is essential for the interaction of Nab2-N with Mlp1-C

**Figure 3.**
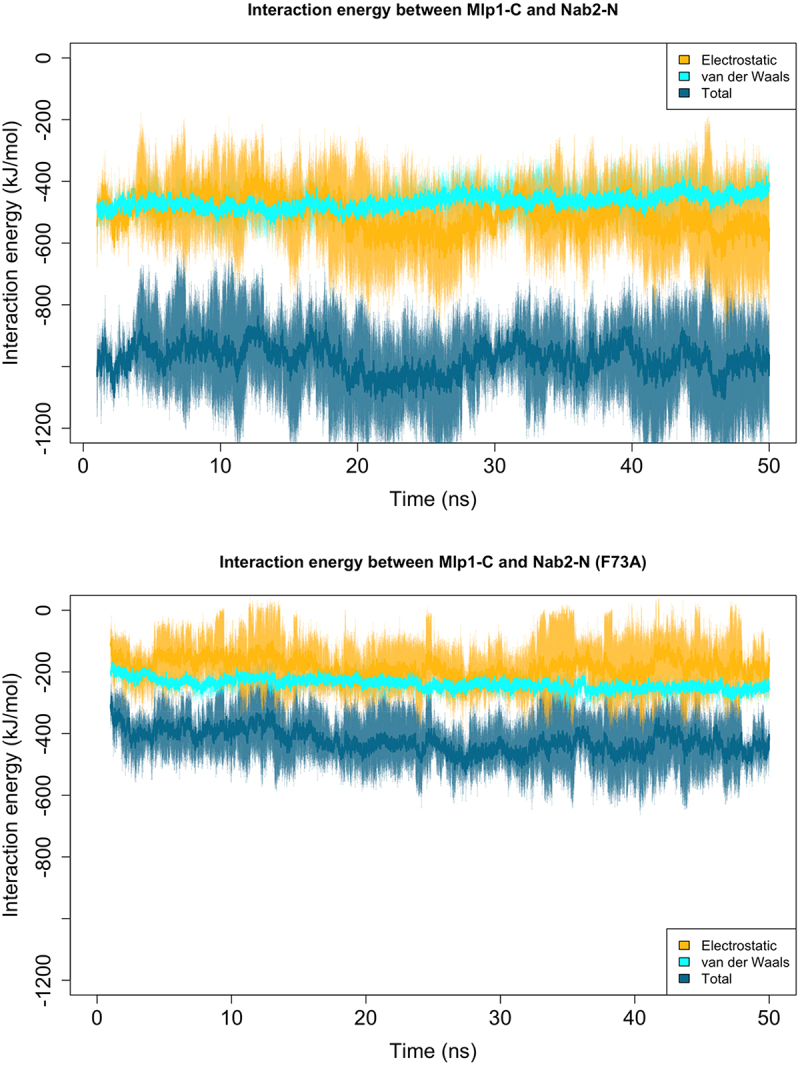
Interaction energy between Mlp1-C and Nab2-N during MD simulations following equilibration. The complex corresponding to docking solution 1 was simulated in wild-type and mutant forms (Nab2-Phe73 mutated to Ala). Each complex (wild-type/mutant) was simulated for 20 ns, with three independent repeats. Solid lines show the mean interaction energy across replicates, and shaded regions indicate variability. The wild-type complex exhibits approximately threefold stronger interaction energy than the mutant, supporting a substantial role of Nab2-Phe73 in this interaction.

To validate our computational protocol and test the significance of Phe73, we compared wild-type Nab2 with an F73A mutant in the solution 1 complex. Because the predicted Mlp1-C structure was partially disordered, we ran 100 ns simulations to ensure equilibration, followed by an additional 20 ns of production runs. Each simulation was repeated three times. These simulations were intended to assess relative stability and mechanistic trends across configurations, rather than to define a unique conformation of the disordered Mlp1-C region. Interaction energies for the wild-type and mutant complexes are shown in Figure 3. The wild-type complex exhibited approximately threefold stronger binding energy compared to the mutant, confirming the essential role of Phe73 and supporting the plausibility of the sampled Mlp1–Nab2 configuration. However, the precise contribution of Phe73 remained unclear. In the following sections, we performed a detailed trajectory analysis to uncover the role of Phe73 in formation of a strong interaction between Nab2-N and Mlp1-C.

### Phe73 plays an indirect role in the interaction between Nab2-N and Mlp1-C

**Figure 4.**
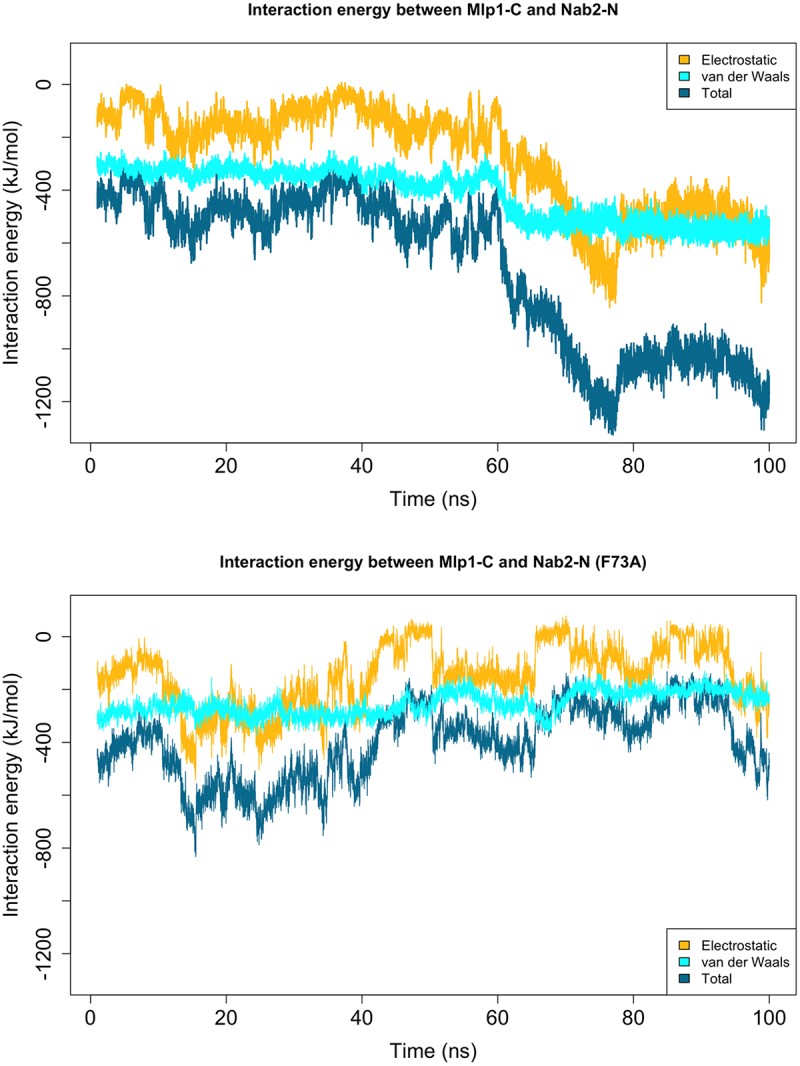
Interaction energy between Mlp1-C and Nab2-N in wild-type (upper) and mutant (lower) simulations during equilibration. Comparison of the two plots indicates that the wild-type complex undergoes a gradual energetic stabilization that is not observed in the mutant, resulting in a stronger interaction.

**Figure 5.**
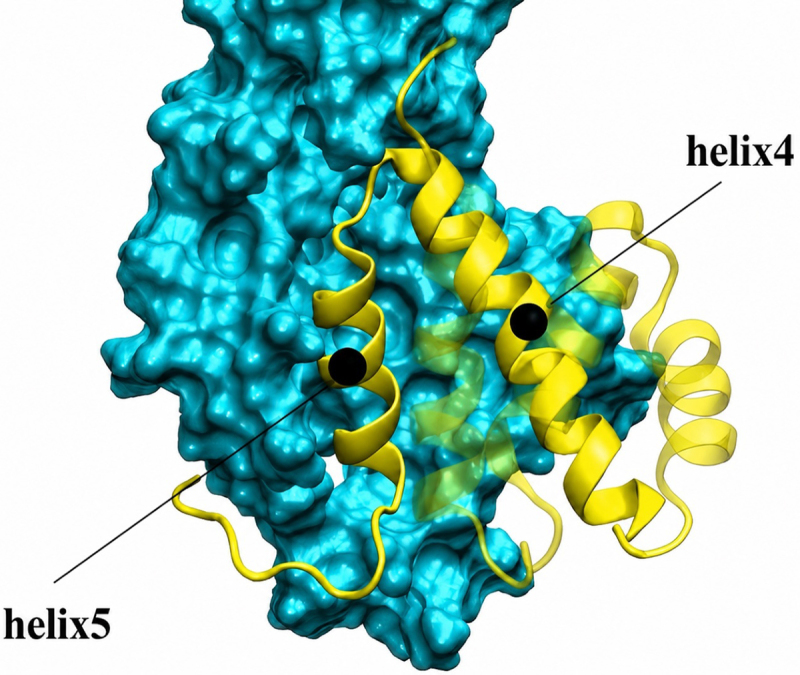
Nab2-N (yellow) consists of five helices, two of which engage with Mlp1-C (cyan) in the simulations. Phe73 of Nab2 is located in the middle of helix4 and forms hydrophobic contacts with the residues on helix5, contributing to stabilization of the helix–helix interface.

Interaction energies were tracked during the 100 ns equilibration (Figure 4). In the wild-type complex, a significant increase occurred at ~60 ns, where the interaction energy rose from ~400 to ~1200 kJ/mol. This was associated with a substantial gain in electrostatic interactions and a modest increase in hydrophobic contacts. On the other hand, the mutant complex showed no such increase, resulting in overall weaker binding. We next measured the direct interaction energy between Phe73 and Mlp1-C, which remained constant at ~40 kJ/mol throughout the simulation (Figure S7). This suggested that Phe73 does not contribute primarily through direct contacts with Mlp1-C. Instead, we examined its role in intramolecular interactions within Nab2-N. Nab2-N comprises five helices, two of which are in direct contact with Mlp1-C. We labeled the Phe73-containing helix as helix4 and the adjacent contacting helix as helix5 (Figure 5).

**Figure 6.**
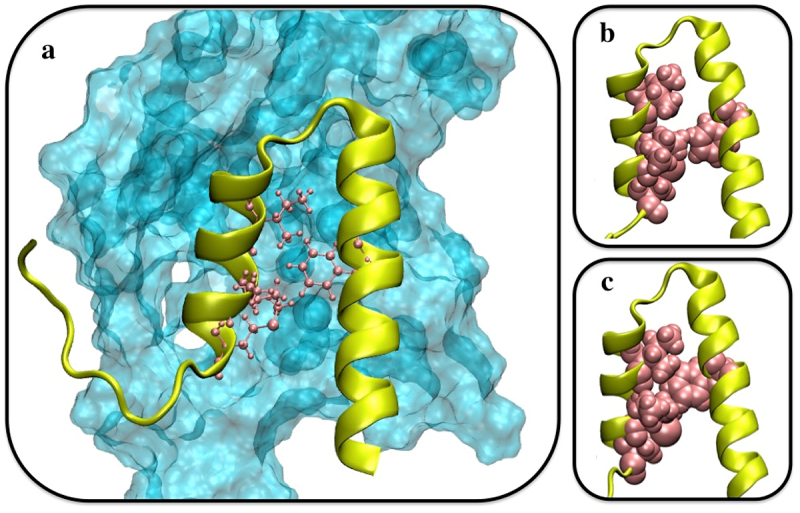
Proposed regulatory role of Phe73 in mediating interactions between helix4 and helix5 of Nab2-N. Mlp1-C is shown in surface representation (transparent cyan) and Nab2-N is shown in yellow with key residues highlighted in pink. Only the two helices of Nab2-N that engage with Mlp1-C in the simulations are displayed. Panels (b) and (c) illustrate representative configurations of these helices prior to and following association with Mlp1-C.

At ~60 ns, the interaction energy between Phe73 and helix5 nearly doubled (Figure S8). Comparative analysis of wild-type and mutant simulations revealed that Phe73 strengthened the helix4–helix5 interface (Figures S9 and S10). The distance between helix4 and helix5 during the production run was maintained at a smaller value in the wild-type (12.61 ± 0.32 Å), compared to that of the mutant (13.51 ± 0.58 Å) (Figure S11). These results suggest that Phe73 functions indirectly by stabilizing the interaction between helix4 and helix5. This intramolecular effect enables both helices to form a stronger composite interface with Mlp1-C. Specifically, the benzyl side chain of Phe73 engages three hydrophobic residues on helix5, effectively locking the helices together and reducing their separation (Figure 6). Mutation of Phe73 to alanine disrupts this mechanism: although alanine is hydrophobic, its small side chain cannot reach the hydrophobic residues on helix5, preventing stabilization of the inter-helix interaction. Phe73 interacts with three hydrophobic residues from helix5. At the point where the drop in the interaction energies is seen (60 ns), the benzyl side chain of Phe73 becomes wedged between these hydrophobic residues, effectively pulling the two helices toward each other, which results in a decrease in the distance mentioned previously. Therefore, the disruptive effect of the F73A mutation can be explained by the distinct side-chain properties: phenylalanine’s bulky benzyl group extends far enough to establish strong hydrophobic contacts with helix5, whereas alanine, despite being hydrophobic, has a much smaller side chain that cannot bridge to helix5 or maintain these stabilizing interactions. This structural difference accounts for the weakened binding observed in the mutant. This hypothesis is in agreement with the experimental observation that F73W mutation not only preserves the strong interaction between Mlp1 and Nab2 but also increases the affinity by a factor of 1.5, as tryptophan also possesses a large benzyl side chain [19]. Our analysis of the F73W mutation also demonstrated that this mutation maintains and strengthens the interaction between the two Nab2 helices, i.e. helix4 and helix5, as well as the Mlp1-Nab2 interaction (Figure S12 and S13). On the other hand, mutating Phe73 to a non-hydrophobic residue significantly weakens the interaction [19] (Figure S14 and S15). Figure 6 illustrates how Phe73 mediates this helix–helix interaction and facilitates complex formation.

In order to further validate the proposed mechanism of interaction, we mutated the three hydrophobic residues on helix5, i.e. I87, I91, and M94, to alanine. The complex of Mlp1-C and mutated Nab2-N was equilibrated for 100 ns and subsequently simulated for an extra 20 ns, which was repeated three times to account for the stochasticity of the system. The complex energy is reduced upon the triple mutation (Figure S16), which further supports our hypothesis on the role of Phe73 and the hydrophobic residues on helix5 in facilitating the Mlp1–Nab2 interaction. However, as expected, the effect of mutating helix5 residues is not as pronounced as F73A, since the latter removes a large benzyl group critical for the binding. Furthermore, the average distance between helix4 and helix5 is measured to be 13.81 ± 0.58 Å, which is closer to the distance after F73A mutation compared to the wild-type.

## Discussion

Mlp1 plays a pivotal role in mRNA quality control at the nuclear basket, where transcripts undergo final surveillance before export [13,18,19]. In this study, we used an integrated computational approach to examine the mechanistic features of Mlp1’s interaction with one of its key partners, Nab2. Understanding this interface is important because Mlp1 binds multiple RBPs during quality control, and its ability to recognize diverse partners underlies its function as a checkpoint protein.

Our results confirm that Phe73 in Nab2 is essential for its interaction with Mlp1 (Figures 3 and 4), while Phe72 does not have a major effect (Figure S17), consistent with earlier experimental studies [18,19]. However, we found that Phe73 does not contribute primarily through direct contacts with Mlp1-C. Instead, it exerts an indirect effect by stabilizing the interactions between helix4 (which contains Phe73) and helix5 of Nab2-N. Strengthened helix–helix packing reduces the distance between these structural elements, resulting in a more compact Nab2-N conformation. This conformational change is compatible with stronger association with the flexible domain of Mlp1-C and the formation of more extensive intermolecular contacts.

**Figure 7.**
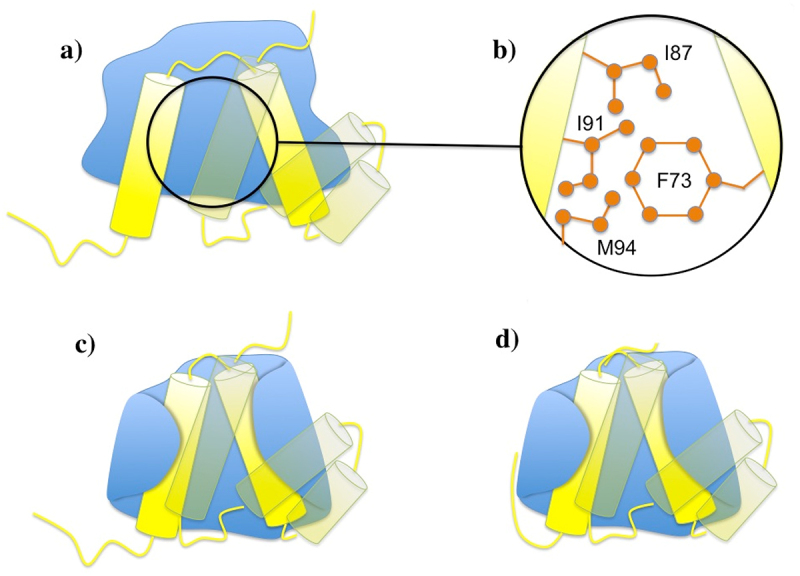
Schematics of a plausible stepwise mechanism for the interaction between Nab2-N and Mlp1-C inferred from docking and MD simulations. a) Nab2-N approaches Mlp1-C as mRNA begins to export through the nuclear pore. b) Nab2-Phe73 forms hydrophobic contacts with residues on helix5 of Nab2-N, c) this interactions reduces the separation between the two helices of Nab2-N that engage with Mlp1-C, facilitating closer association with the flexible, intrinsically disordered region Mlp1-C, d) the coil at the end of Nab2-N can then reposition to form additional electrostatic contacts with Mlp1-C. Only the predicted disordered region of Mlp1-C is shown.

Based on these findings, we propose a plausible stepwise mechanism for Nab2–Mlp1 binding during mRNA quality control: 1) mRNA-bound Nab2 approaches the nuclear basket where Mlp1 is located (Figure 7(a)); 2) In the vicinity of Mlp1-C, Nab2-Phe73 approaches helix5 of Nab2. Our results show that, in the absence of Mlp1, it is more favorable for Nab2-Phe73 to move away from helix5 rather than approaching it (Figure S18), suggesting that the presence of Mlp1 biases this interaction (Supplementary Materials section E); 3) Phe73 engages three hydrophobic residues on helix5 (I87, I91, and M94), which reduces the inter-helical distance and compacts Nab2-N (Figure 7(b)); 4) As a result of the reduced distance between the two helices of Nab2-N, the more compact Nab2-N comformation facilitates closer engagement with the flexible C-terminal domain of Mlp1-C, leading to enhanced hydrophobic interactions (Figure 7(c)); 5) Subsequent rearrangement of the charged loop at the end of Nab2-N enables additional electrostatic interactions to further stabilize the complex (Figure 7(d)) (Figure S19).

We note that this model is based on simulations of Nab2-N (residues 1–97) in isolation, and that inclusion of additional Nab2 domains, mRNA, or export receptors could alter the binding trajectory. Although the adjacent RGG domain is largely disordered and unlikely to impose major steric constraints, such factors may influence later stages of complex formation. Accordingly, the final electrostatic ‘lock’ step should be interpreted cautiously and validated experimentally. Notably, the mutant complex (F73A) did not form these late-stage electrostatic interactions, underscoring the regulatory importance of Phe73 (Figure 4). Additionally, because Mlp1-C contains an intrinsically disordered region, our docking and simulations should be interpreted as identifying a plausible binding mechanism and representative interaction modes, rather than a unique structure.

Our findings provide mechanistic insight and a testable computational model for the Mlp1–Nab2 interaction and help frame the broader role of Mlp1 as a multi-partner checkpoint protein in mRNA export and quality control. The C-terminal domain of Mlp1 has been suggested to function as a docking site for mRNPs through interactions with multiple RBPs [13,18,22]. Although still speculative, our proposed ‘wrap-around’ model offers a possible explanation for how the structural flexibility of Mlp1’s disordered C-terminal domain enables adaptive recognition of diverse RBPs by responding to their conformational states. This flexibility may represent a general strategy by which the nuclear basket accommodates and inspects mRNPs. Future experimental and computational studies will be needed to test whether this model extends to additional RBPs and to fully define the spectrum of Mlp1’s interactions at the nuclear pore.

## Supplementary Material

Supplementary_materials_revision_Clean copy.docx

## Data Availability

All data supporting this study are available as follows: Docking inputs/poses, MD input files, parameter/topology files, R scripts to generate the figures, and representative trajectories are deposited at Zenodo (https://doi.org/10.5281/zenodo.19503386). The starting Nab2 N-terminal structure is PDB 2V75; predicted Mlp1-C models and final coordinates for WT and mutants are included. Additional data can be provided upon request.
