# Supplementary material for: Intrinsically disordered regions facilitate Mlp1–Nab2 recognition in mRNA quality control
Source: Nucleus. 2026 Jun 3;17(1):2680376. doi: 10.1080/19491034.2026.2680376 (PMC13240961; doi:10.1080/19491034.2026.2680376)
Supplement: Supplementary_materials_revision_Clean copy.docx [file KNCL_A_2680376_SM2659.docx]

*Supplementary materials for*

**Intrinsically disordered regions facilitate Mlp1–Nab2 recognition in mRNA quality control**

M. Soheilypour, M. Peyro, H. Shams, MRK Mofrad*

The Nab2-binding domain of Mlp1 was mapped to a 183-residue region within its C-terminal domain (Mlp1-C; residues 1586-1768). Since no experimental structure is available for this region, we used various secondary and 3D structure prediction tools, including Phyre2, I-Tasser, RaptorX, Psi-Pred, and HHPred [1–5], to obtain representative structural models of Mlp1-C. I-Tasser predicted that the first 84 residues form helices, while the remaining residues are largely disordered (with two very small helices). The confidence score is calculated to be -2.06 in a [-5, 2] range, and the TM-score is 0.47±0.15, where TM>0.5 typically indicates correct topology [6]. Phyre2 similarly predicted helical structure for the first 86 residues and a predominantely disordered C-terminal segment, with 70% confidence for the helical region [1]. Consistent results were obtained from Psipred [3], RaptorX [2], and HHpred, all of which identified two helices followed by an extended disordered region. Therefore, while the precise conformation of the disordered portion remains uncertain, there is strong agreement that Mlp1-C comprises a short helical segment and a flexible C-terminal domain. The predicted overall organization is in agreement with previous reports on Tpr (human homologue of Mlp1) structure, which has a long coiled coil region followed by a disordered domain at the C-terminus [7]. In this study, the Phyre2 model is used as a representative starting conformation of Mlp1-C for docking and MD simulations (Figure S1).


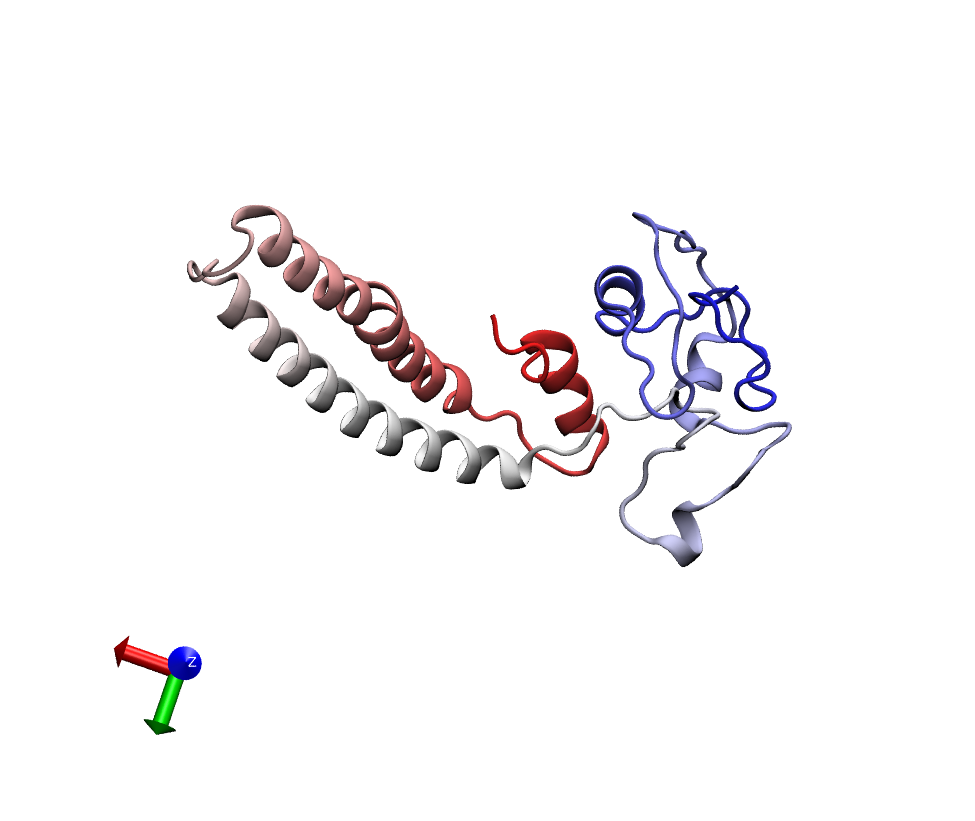

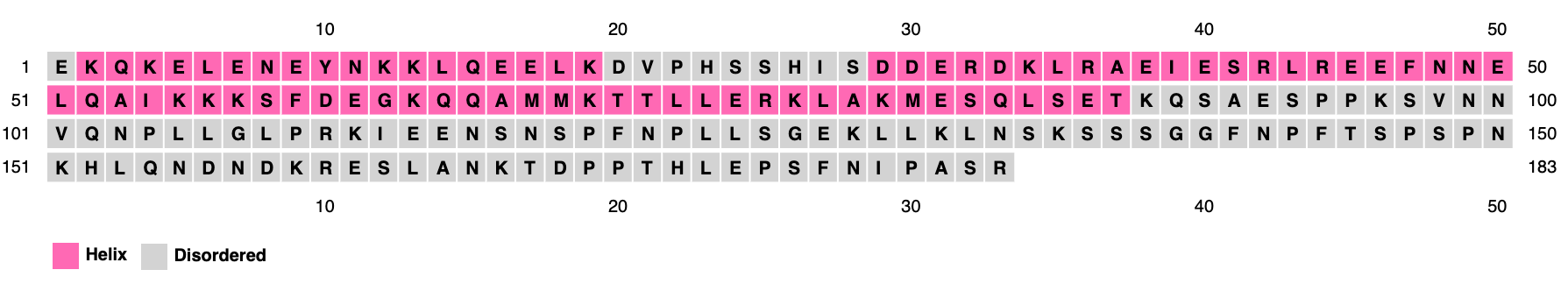


Figure S1: Representative predicted model of the C-terminal domain of Mlp1 (residues 1586-1768) generated using the Phyre2 Web server (1). The structural model is colored from the N-terminus (red) to the C-terminus (blue). Consistent with all prediction tools used, the N-terminal domain forms two helices, while the remainder is predicted to be largely disordered. The aligned sequence shown below the structure highlights the correspondence between predicted secondary structure and amino acid composition. This region contains no FG repeats, consistent with the low FG content reported for the Mlp1/Tpr family, including the limited number of FG motifs observed in mammalian Tpr. This model is used as a representative starting conformation, and not as a definitive structural solution for the intrinsically disordered region.

There is no structural information available for the Mlp1-Nab2 complex. Therefore we used molecular docking (ZDOCK server [8]) to explore a range of plausible interaction modes consistent with available experimental constraints. Prior to docking, both Mlp1-C and Nab2-N were equilibrated for 20 ns to relax any modeling artifacts. Across the top 500 docking poses, we observed a strong enrichment of Nab2-N orientations near the predicted disordered region of Mlp1-C (Figure S2). This pattern suggests that the structured helical segment of Mlp1-C is unlikely to serve as the docking site, as very few poses positioned Nab2-N in that region.


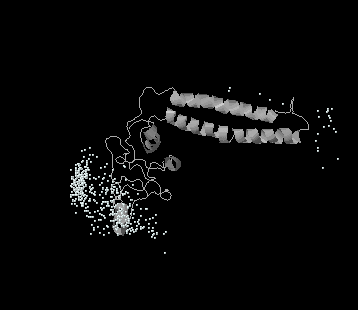


Figure S2: Distribution of the top 500 ZDOCK solutions for the Mlp1-C and Nab2-N interaction. The full Mlp1-C model is shown, while only centers of mass of Nab2-N in each solution are presented by dots. The concentration of solutions around the predicted disordered region of Mlp1-C indicates that this flexible segment is frequently compatible with sampled docking poses for Nab2-N.

To evaluate whether the initial conformation of the Mlp1-C disordered region remained suitable as a starting point for docking, we monitored its behavior during the 20 ns equilibration. Although intrinsically disordered regions are expected to sample multiple conformations, the overall solvent accessible surface area (SASA) (Figure S3) and backbone root mean square deviation (RMSD) (Figure S4) stabilized without large-scale rearrangements over this interval. This indicates that the chosen model remained structurally **representative for initiating docking calculations**, while not implying a single defined conformation for the disordered region in solution.


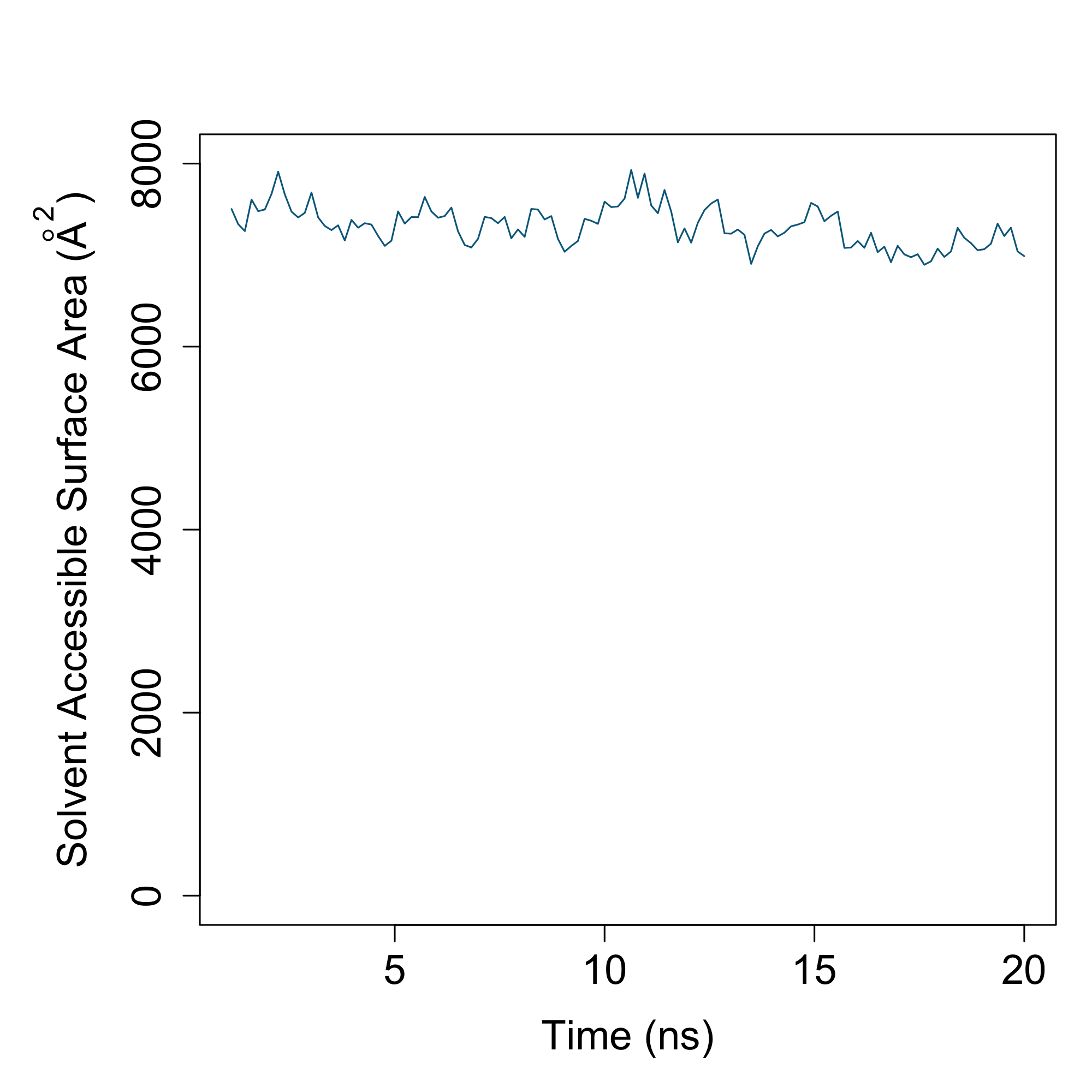


Figure S3: Solvent accessible surface area (SASA) of the predicted disordered region of Mlp1-C during a 20 ns equilibration. No large-scale changes were observed over this timescale, suggesting that the chosen conformer remained a reasonable representative of the ensemble for initiating docking.


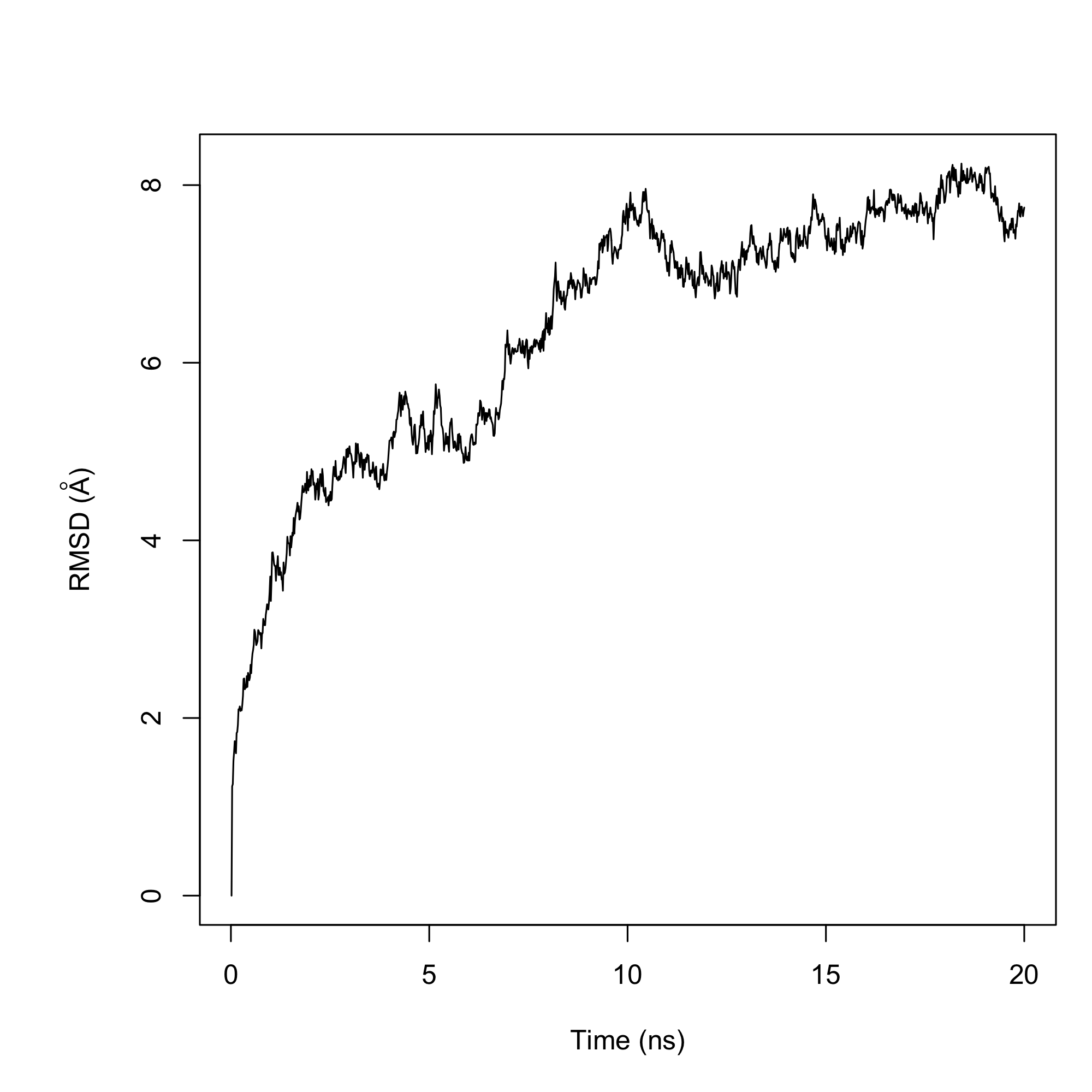


Figure S4: Root mean square deviation (RMSD) of the predicted disordered region of Mlp1-C during equilibration. The RMSD reaches a plateau, indicating stabilization of the initial representative conformer for the purposes of docking, without implying a single fixed structure in solution.


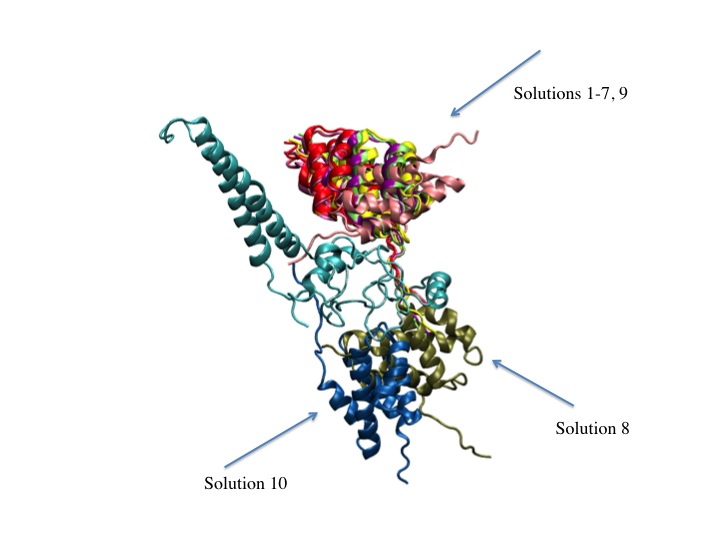


Figure S5: Patchdock and Firedock docking results. Only one of the solutions (solution 8) out of the first 10 solutions is compatible with the fact that Nab2-Phe73 is crucial for the interaction. Solution 8 is identical to the most favorable solution from ZDock server (solution 1).


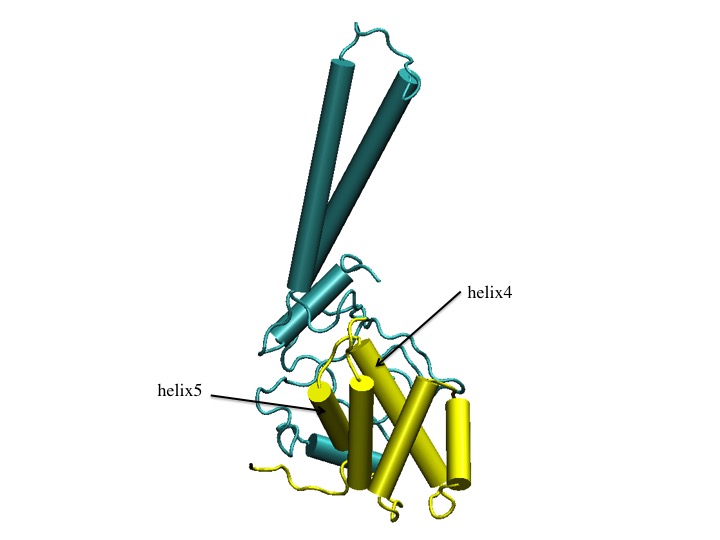


Figure S6: Simple cartoon of the most favorable predicted orientation of interaction. The disordered domain of Mlp1-C engages with helices 4 and 5 of Nab2-N.


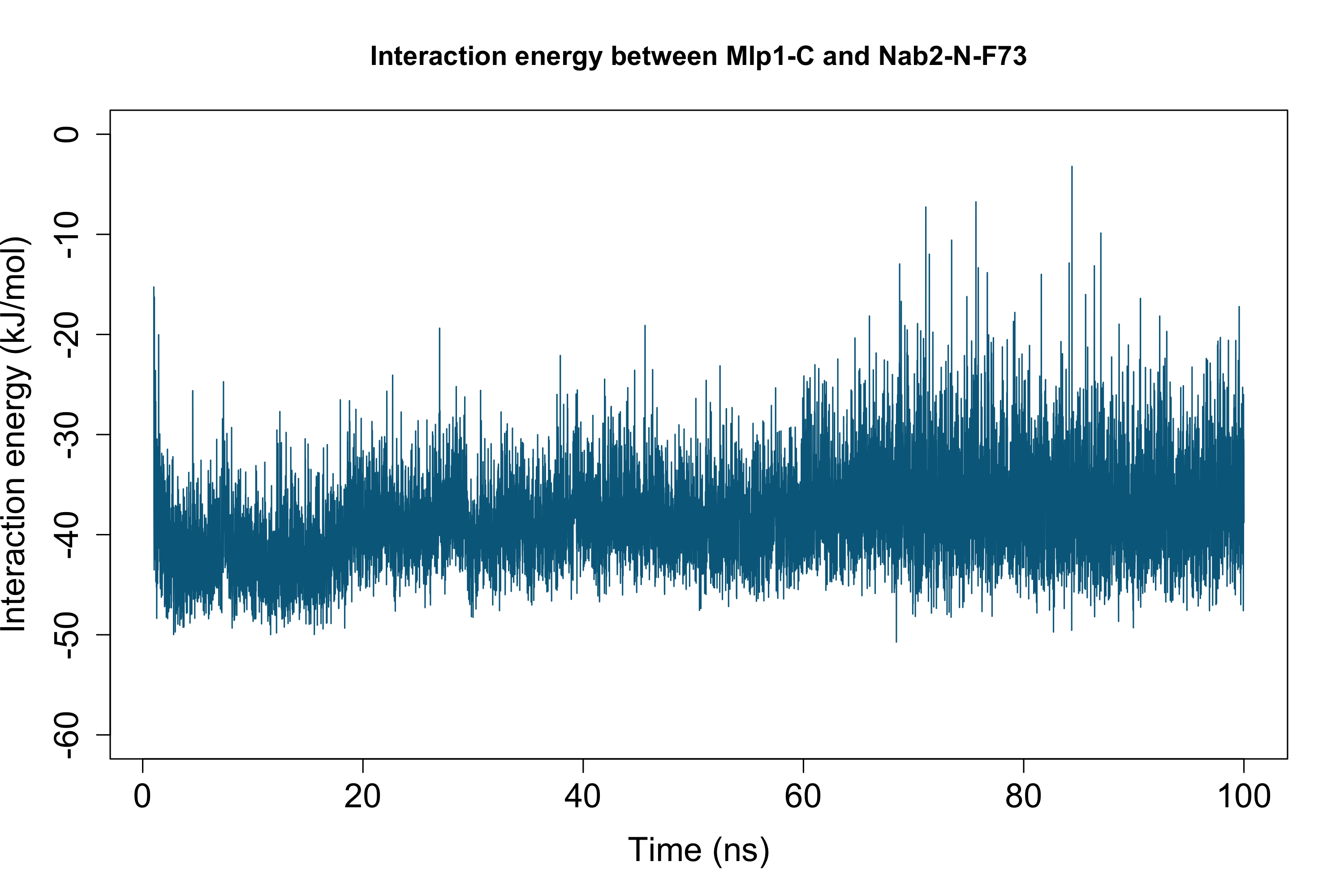


Figure S7: Interaction energy between Mlp1-C and Phe73 of Nab2-N. The graph shows almost constant hydrophobic interaction between the two, which suggests that the increase in interaction energy between Nab2-N and Mlp1-C does not happen through engagement of Phe73 with Mlp1-C, and there are probably other mechanisms involved.


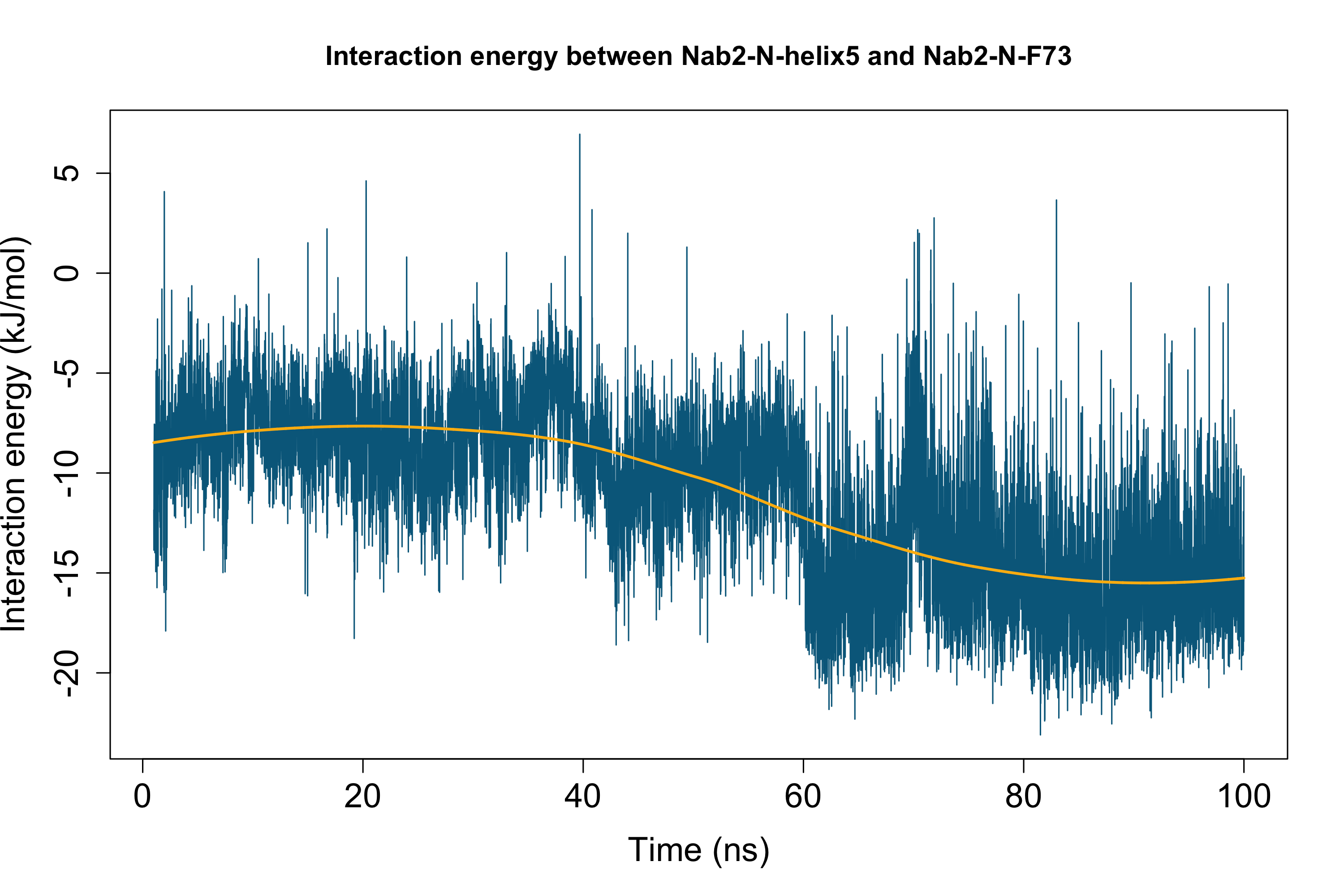


Figure S8: The interaction energy between Phe73 and Nab2-N-helix5. This energy plot indicates that most likely Phe73 affects binding between Mlp1-C and Nab2-N through its interaction with helix5 of Nab2-N.


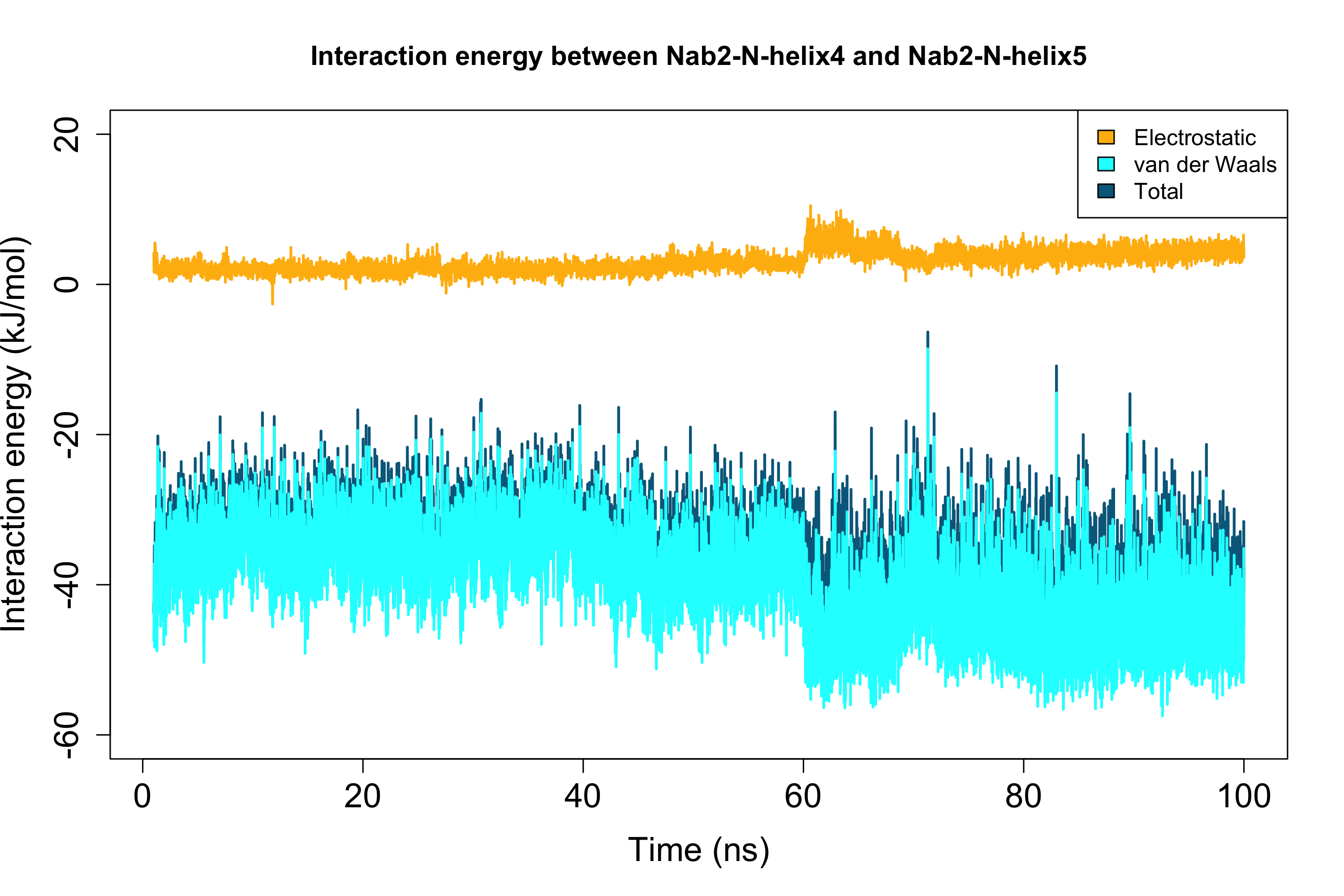


Figure S9: The interaction energy between the two helices of Nab2-N. At 60ns, the hydrophobic interaction between the two helices slightly increases.


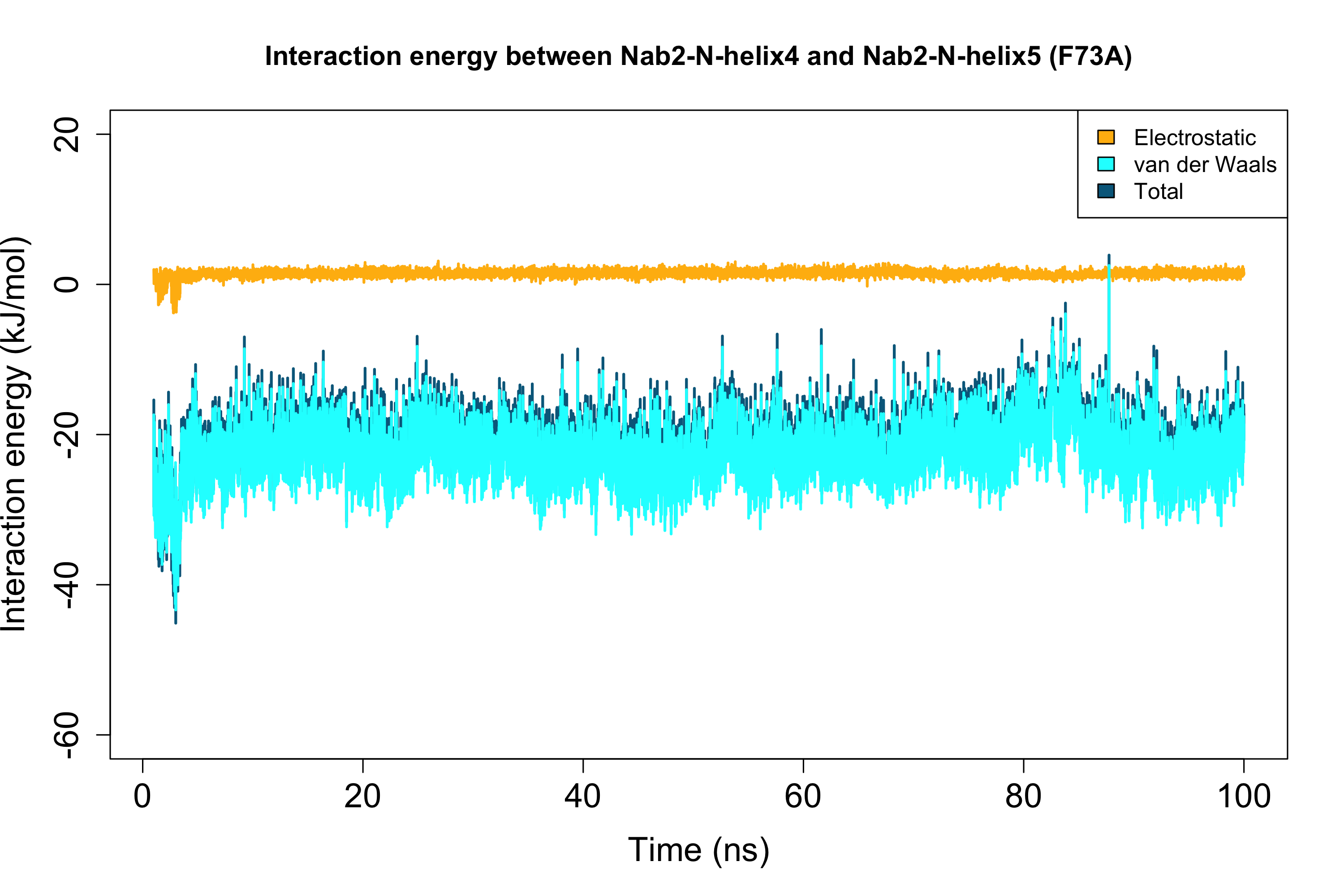


Figure S10: The interaction energy between the two helices of Nab2-N in the mutant.


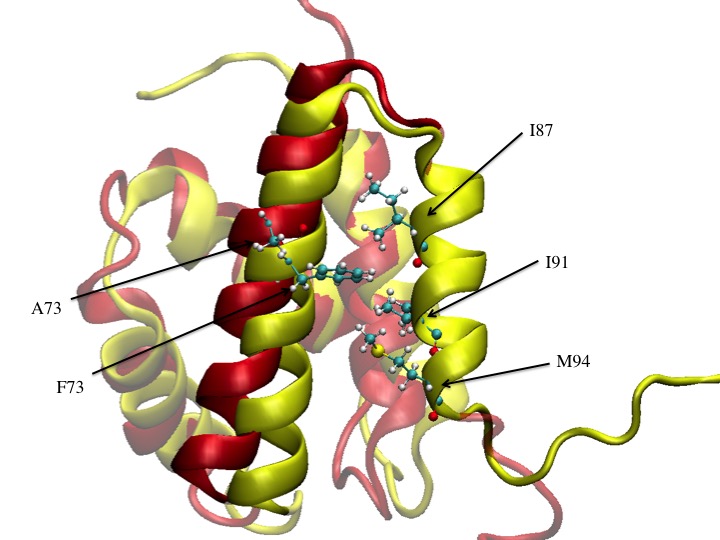


Figure S11: Structural context of Nab2-N point mutations analyzed in this study. The interaction between hydrophobic residues of helix4 and helix5 of Nab2-N is shown for the wild-type (yellow) and the F73A mutant (red). Phe73, located at the center of helix4, engages three hydrophobic residues on helix5 (I87, I91, and M94), forming a stabilizing inter-helical interface. Mutation of Phe73 to alanine disrupts these contacts and increases the distance between the two helices. This figure highlights the location of the Nab2-N point mutation within the folded N-terminal domain and its immediate structural environment.


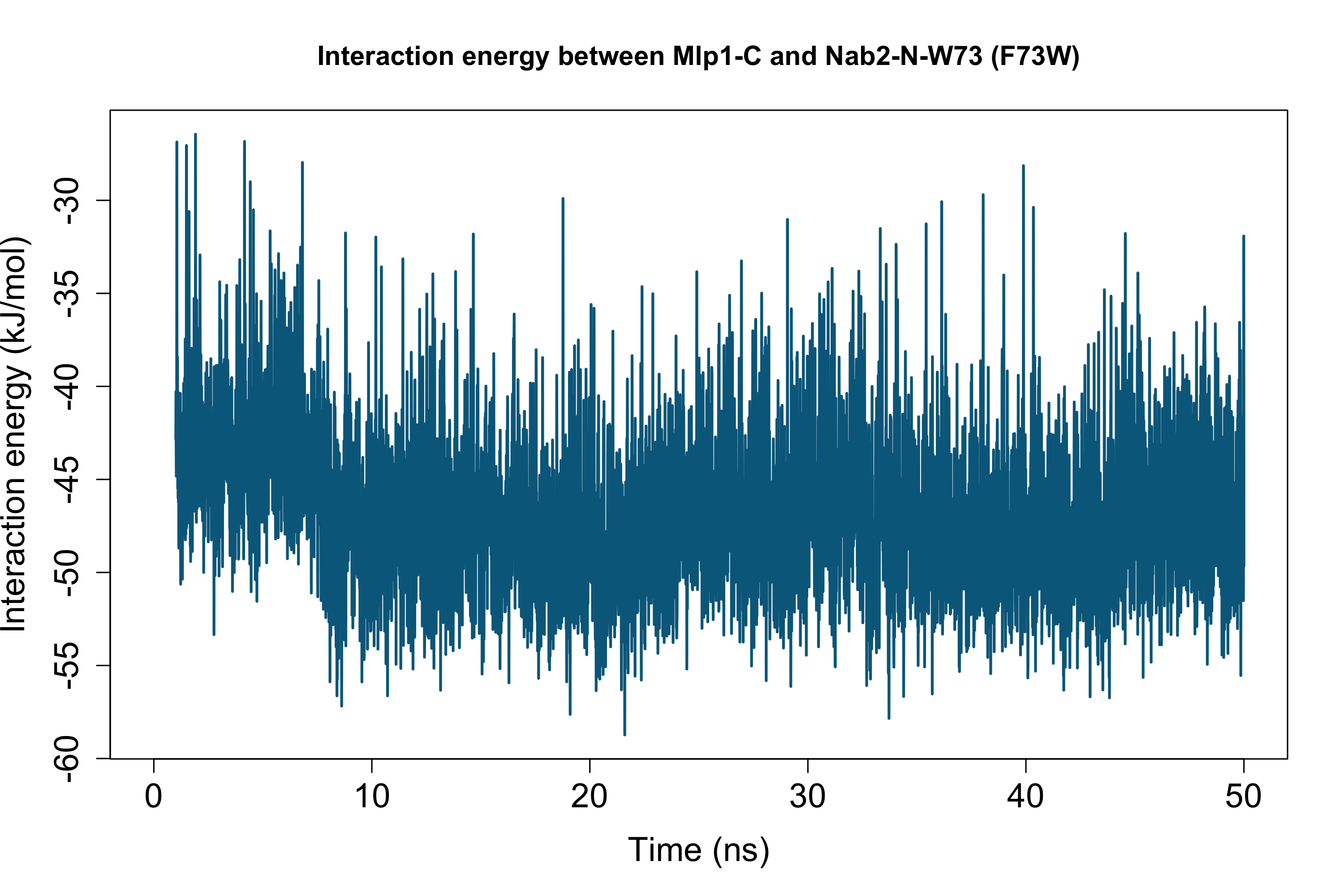


Figure S12: The interaction energy between Mlp1-C and Nab2-N-W73. The more stable and stronger interaction energy of Nab2-N-W73 indicates that F72W mutation strengthens the Mlp1-Nab2 interaction.


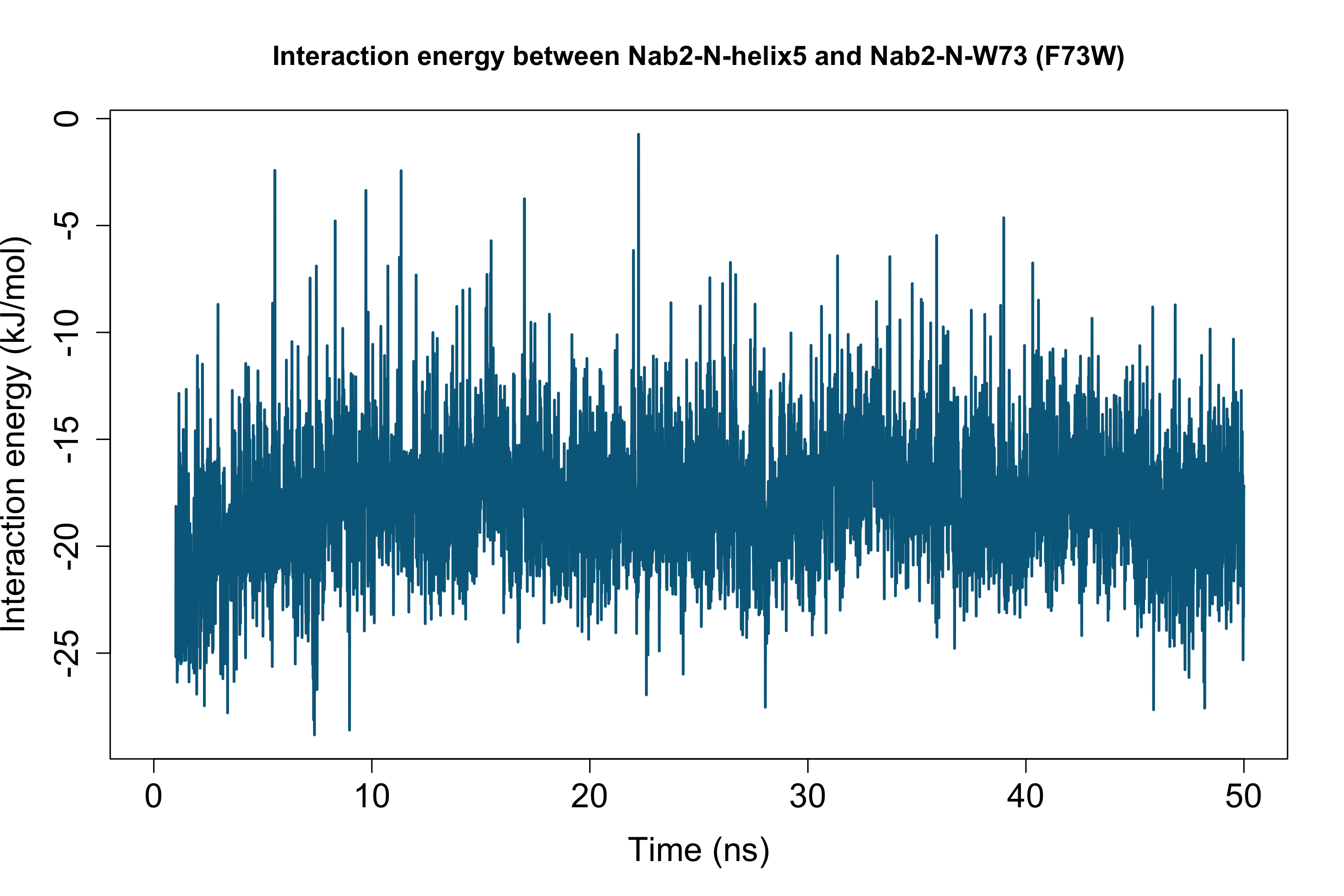


Figure S13: The interaction energy between Nab2-N-helix5 and Nab2-N-W73. The interaction energy indicates that F72W maintains and strengthens the interaction between the two helices as well.


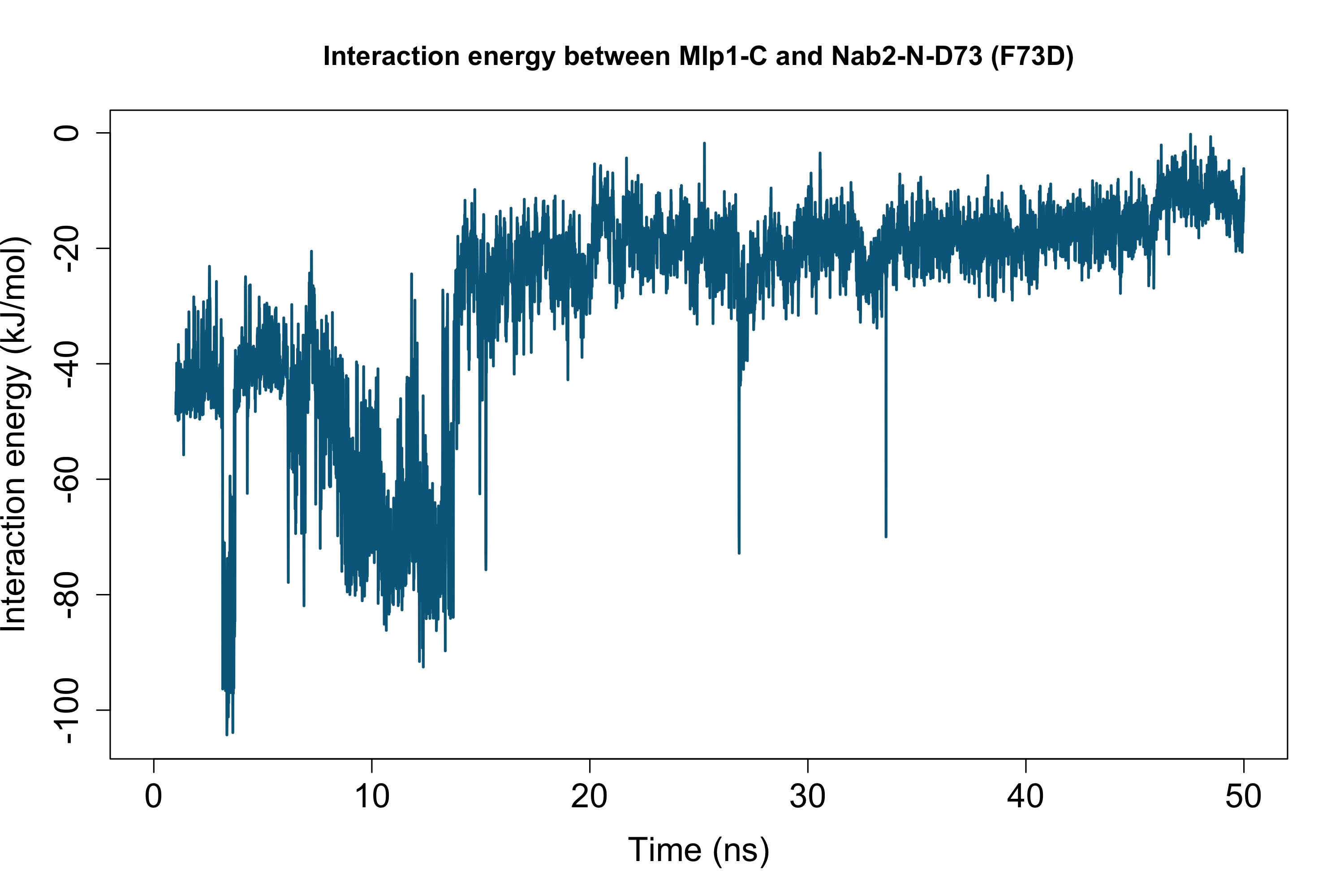


Figure S14: The interaction energy between Mlp1-C and Nab2-N-D73. The interaction energy is dramatically reduced, demonstrating that F73D significantly weakens Mlp1-Nab2 interaction.


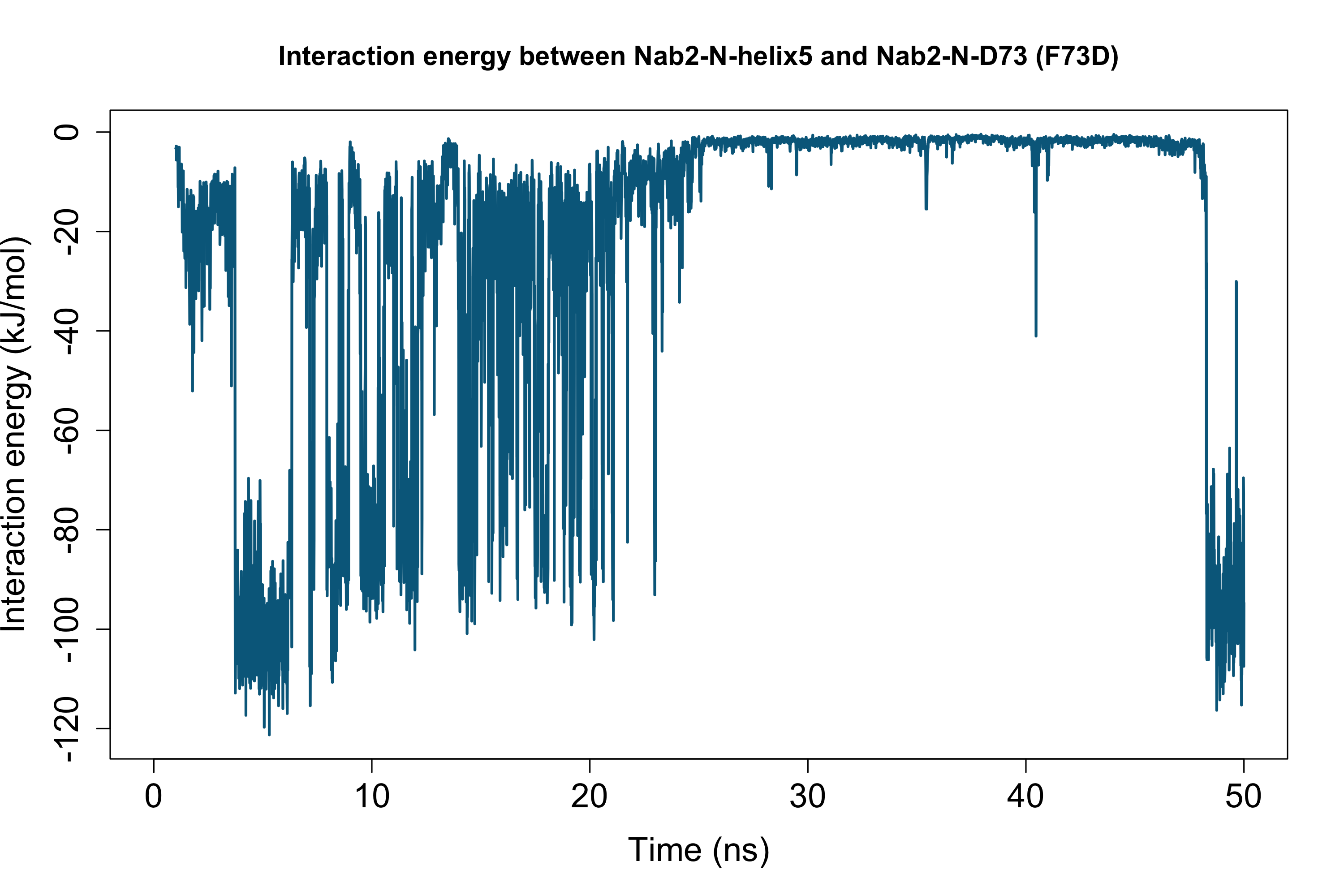


Figure S15: The interaction energy between Nab2-N-helix5 and Nab2-N-D73. The interaction between helices becomes markedly unstable upon F73D, as expected.


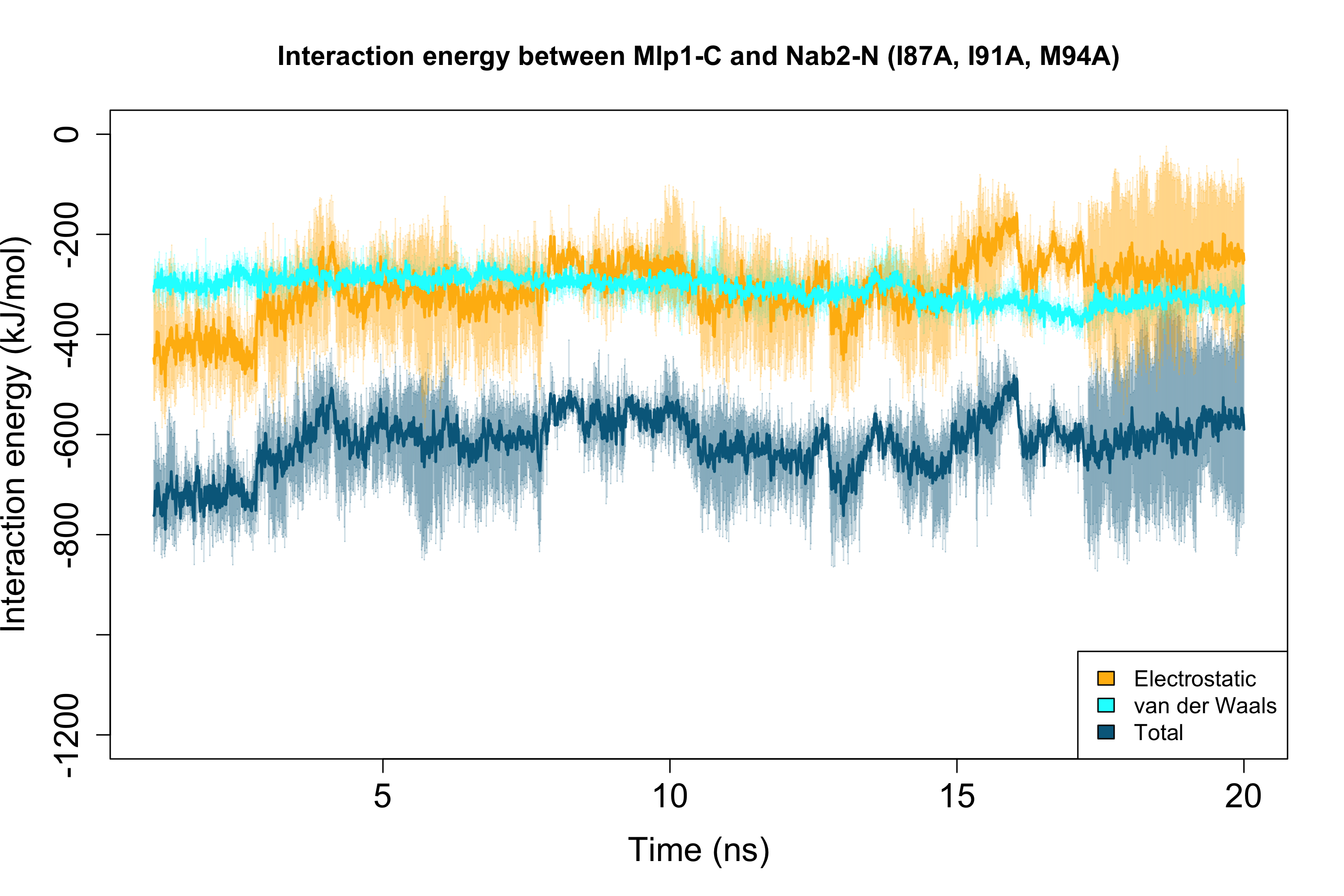


Figure S16: The interaction energy between Mlp1-C and mutated residues on helix5 of Nab2-N namely I87A, I91A, and M94A.


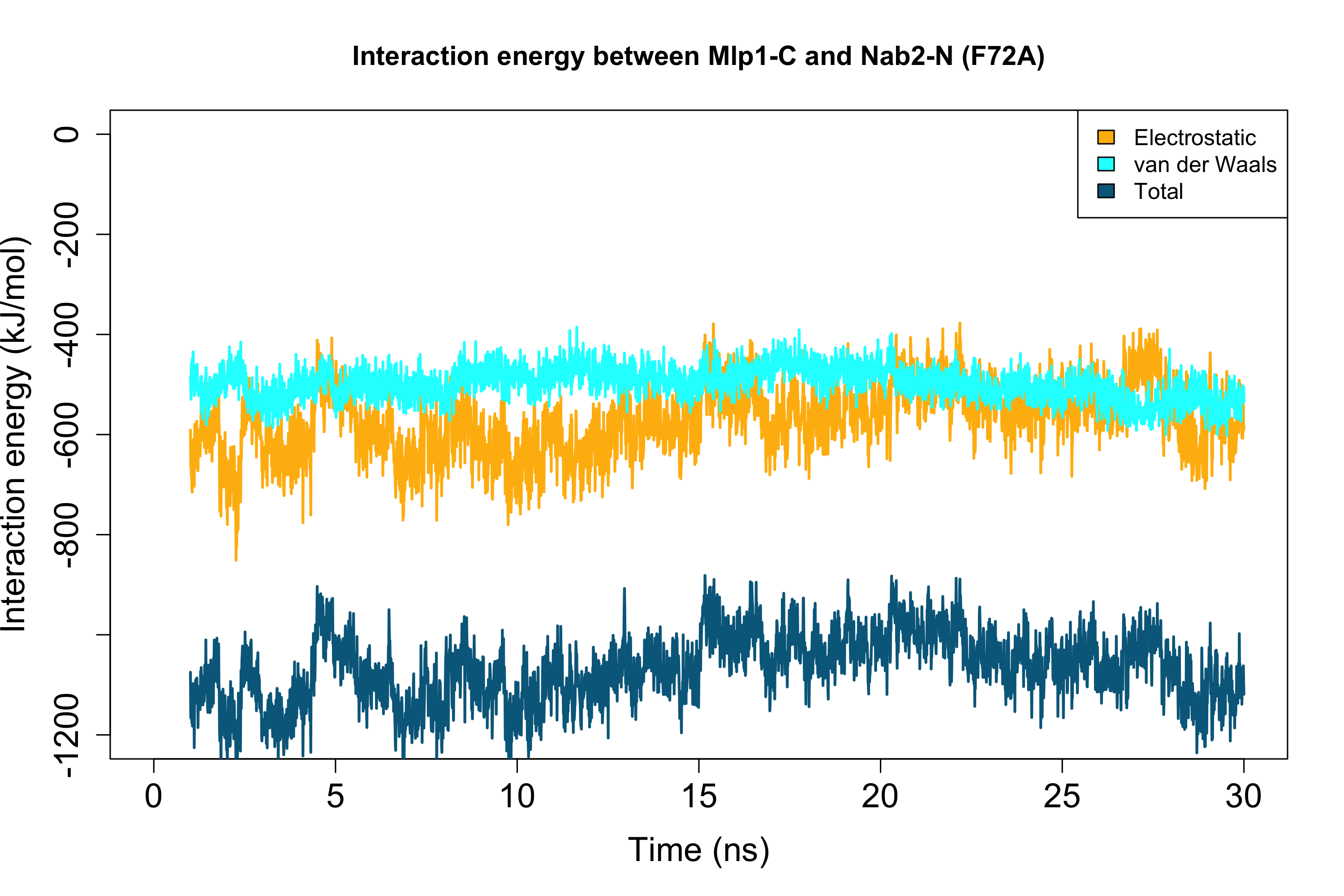


Figure S17: The interaction energy between Mlp1-C and Nab2-N-F72A. The stable interaction energy indicates that F72 does not have a major effect on the interaction, as was observed experimentally by Fasken et al (2008).

We isolated Nab2 from the middle frame of the binding simulation between Mlp1-C and Nab2-N to examine how Phe73 behaves in the absence of Mlp1. At the beginning of the 1 ns simulation, Phe73 was engaged with the hydrophobic residues of helix5 (Figure S19-a). However, after about 0.1 ns, Phe73 rotated and pointed outward (Figure S19-b). This indicates that strong engagement between Phe73 and helix5 preferentially occurs in the vicinity of Mlp1 and is diminished in its absence.


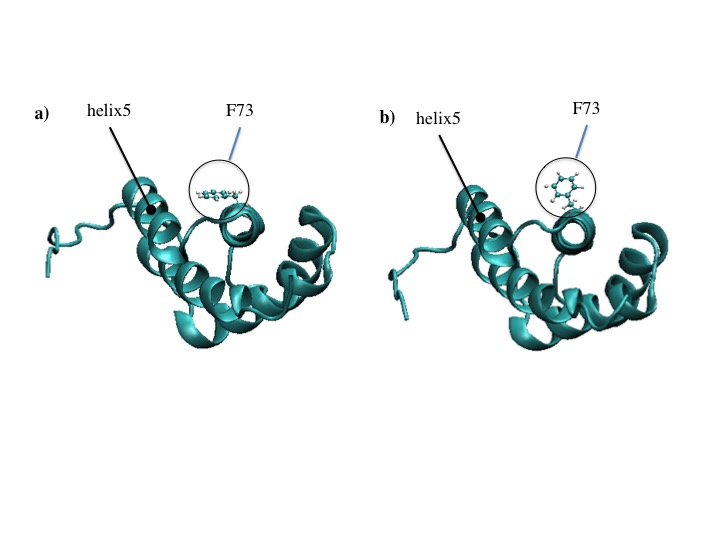


Figure S18: The presence of Mlp1-C is required for Nab2-Phe73 to be able to approach helix5. a) Nab2-Phe73 is engaged with helix5 in the bound state to Mlp1, while b) the side chain of Nab2-Phe73 is rotated outward once Mlp1-C is eliminated from the simulation.


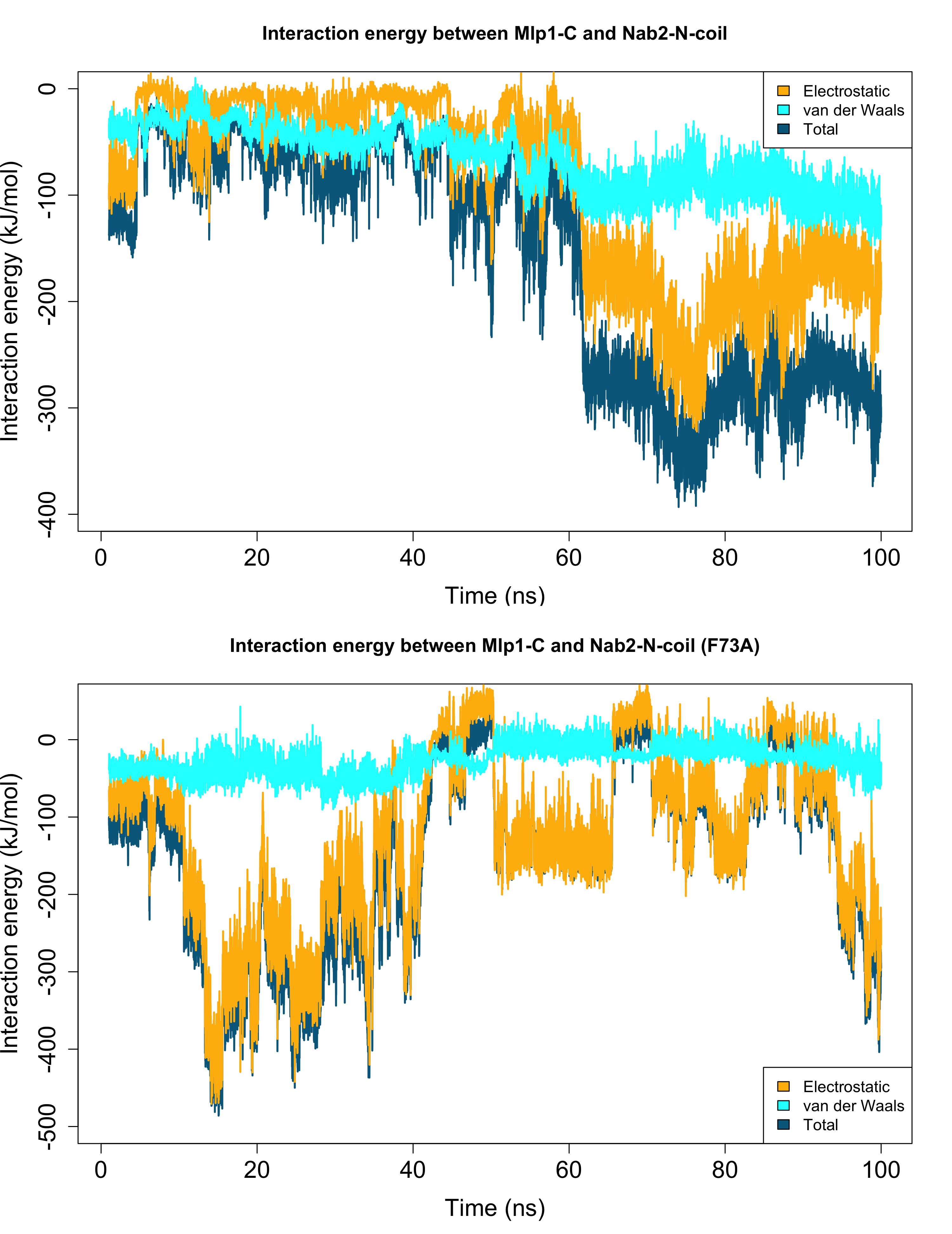


Figure S19: The interaction energy between the coil at the end of Nab2-N with Mlp1-C in a) the wild-type (upper plot) and b) mutant (lower plot). The coil at the end of Nab2-N forms a sustained electrostatic interaction with Mlp1-C in the wild-type.

**References**

1. Kelley LA, Sternberg MJE. Protein structure prediction on the Web: a case study using the Phyre server. Nat Protoc. 2009 Feb;4(3):363–71.

2. Wang Z, Zhao F, Peng J, Xu J. Protein 8-class secondary structure prediction using conditional neural fields. Proteomics. 2011 Oct;11(19):3786–92.

3. McGuffin LJ, Bryson K, Jones DT. The PSIPRED protein structure prediction server. Bioinformatics. 2000 Apr 1;16(4):404–5.

4. Roy A, Kucukural A, Zhang Y. I-TASSER: a unified platform for automated protein structure and function prediction. Nat Protoc. Nature Publishing Group; 2010 Apr;5(4):725–38.

5. Söding J. Protein homology detection by HMM-HMM comparison. Bioinformatics. 2005 Apr 1;21(7):951–60.

6. Zhang Y, Skolnick J. Scoring function for automated assessment of protein structure template quality. Proteins. 2004 Dec 1;57(4):702–10.

7. Byrd DA, Sweet DJ, Panté N, Konstantinov KN, Guan T, Saphire AC, et al. Tpr, a large coiled coil protein whose amino terminus is involved in activation of oncogenic kinases, is localized to the cytoplasmic surface of the nuclear pore complex. J Cell Biol. 1994 Dec;127(6 Pt 1):1515–26.

8. Pierce BG, Wiehe K, Hwang H, Kim B-H, Vreven T, Weng Z. ZDOCK server: interactive docking prediction of protein-protein complexes and symmetric multimers. Bioinformatics. 2014 Jun 15;30(12):1771–3.
